# Systemic inflammatory response syndrome and prolonged hypoperfusion lesions in an infant with respiratory syncytial virus encephalopathy

**DOI:** 10.1007/s10156-013-0558-0

**Published:** 2013-01-26

**Authors:** Kenji Miyamoto, Masahide Fujisawa, Hajime Hozumi, Tatsuo Tsuboi, Shigeko Kuwashima, Jun-ichi Hirao, Kenichi Sugita, Osamu Arisaka

**Affiliations:** 1 Department of Pediatrics, Dokkyo Medical University, 880 Kitakobayashi, Mibu-machi, Shimotsuga, Tochigi, 321-0293 Japan; 2 Department of Radiology, Dokkyo Medical University, Tochigi, Japan; 3 Clinical Training Center, Dokkyo Medical University, Tochigi, Japan

**Keywords:** Respiratory syncytial virus, Encephalopathy, Cytokine, Infant, Single-photon emission computed tomography

## Abstract

Respiratory syncytial virus (RSV) is a cause of neurological complications in infants. We report a rare case of RSV encephalopathy in an infant who presented with poor sucking and hypothermia at 17 days of age after suffering from rhinorrhea and a cough for several days. After hospitalization, the patient presented with stupor and hypotonia lasting for at least 24 h, and was intubated, sedated, and ventilated for treatment of pneumonia. These symptoms led to diagnosis of pediatric systemic inflammatory response syndrome (SIRS) caused by RSV infection. High-dose steroid therapy was combined with artificial ventilation because the initial ventilation therapy was ineffective. Interleukin (IL)-6 levels in spinal fluid were markedly increased upon admission, and serum IL-6 and IL-8 levels showed even greater elevation. The patient was diagnosed with RSV encephalopathy. On day 5, high signal intensity in the bilateral hippocampus was observed on diffusion-weighted magnetic resonance imaging (MRI). On day 14, the patient presented with delayed partial seizure and an electroencephalogram showed occasional unilateral spikes in the parietal area, but the hippocampal abnormality had improved to normal on MRI. ^99m^Tc-labeled ethylcysteinate dimer single-photon emission computed tomography (SPECT) on day 18 showed hypoperfusion of the bilateral frontal and parietal regions and the unilateral temporal region. SPECT at 3 months after onset still showed hypoperfusion of the bilateral frontal region and unilateral temporal region, but hypoperfusion of the bilateral parietal region had improved. The patient has no neurological deficit at 6 months. These findings suggest that RSV encephalopathy with cytokine storm induces several symptoms and complications, including SIRS and prolonged brain hypoperfusion on SPECT.

## Introduction

Infection with respiratory syncytial virus (RSV) causes bronchitis, bronchiolitis, and pneumonia, and leads to neurological complications in 1.8 % of cases [1]. These neurological complications include RSV encephalopathy [2, 3], the cause of which remains unclear. Kawashima et al. [4] defined three pathogenic types of RSV encephalopathy characterized by metabolic error, excitotoxicity, and cytokine storm. Here, we describe a rare case of a neonate with RSV encephalopathy of the cytokine storm type, which involved development of pediatric systemic inflammatory response syndrome (SIRS) and prolonged hypoperfusion lesions on brain single-photon emission computed tomography (SPECT) without neurological sequelae.

## Case report

The patient was a male infant born at 39 weeks gestation (birth weight, 3,562 g). At this time, his brother and cousin both had a cold, and the patient presented with rhinorrhea and cough after several days. At 17 days of age, his parents brought him to the emergency department because of poor sucking for more than half a day and hypothermia (33.0 °C). The patient was in a continuous stupor with hypotonia and did not respond to pain. He presented with cyanosis, and percutaneous oxygen saturation was 86 %. The desaturation was reversed by oxygenation. After hospitalization, a physical examination showed wheezes and hepatomegaly. Respiratory therapy with nasal continuous positive airway pressure was started, but apnea persisted.

Laboratory findings upon admission showed increased aspartate aminotransferase (AST), alanine aminotransferase (ALT), lactase dehydrogenase (LDH), creatine phosphokinase (CPK), and carbon dioxide (PaCO_2_) (Table 1). AST, ALT, LDH, and CPK levels in serum increased progressively until day 2. RSV antigen was detected in a nasopharyngeal swab. The results of several cultures were negative. Chest radiography and computed tomography scans showed bilateral pulmonary infiltrative shadow, atelectasis, and severe air trapping. A diagnosis of pediatric SIRS caused by RSV infection was made based on the published criteria for this condition [5].

**Table 1:** Laboratory data at admission

Peripheral blood		Arterial blood gas analysis (FiO_2_ 0.30–0.45)	
WBC	20,200/μl	pH	7.228
Nt	31 %	PaCO_2_	69.4 Torr
Ly	47 %	PaO_2_	72.7 Torr
Mo	10 %	HCO_3_^−^	27.9 mmol/l
Eo	2 %	BE	−0.1 mEq/l
Ba	0 %	Serology	
RBC	305 × 10^4^/μl	CRP	3.87 mg/dl
Hb	10.1 g/dl	Procalcitonin	0.77 ng/ml
Ht	32.4 %	IgG	853 mg/dl
Plt	52.7 × 10^4^/μl	IgM	32 mg/dl
Biochemistry		IgA	5 mg/dl
TP	6.7 g/dl	Ferritin	8163 ng/ml
Alb	4.1 g/dl	IL-6 (<4 pg/ml)^a^	304 pg/ml
AST	192 U/l	Serology during hospitalization	
ALT	160 U/l	IL-1β (<10 pg/ml)^a^	<200 pg/ml
T-bil	1.3 mg/dl	IL-8 (<2 pg/ml)^a^	115 pg/ml
LDH	1359 U/l	IL-10 (<5 pg/ml)^a^	<20 pg/ml
CK	439 U/l	MCP-1 (200–722 pg/ml)^a^	<625 pg/ml
BUN	12 mg/dl	TNF-α (0.6–2.8 pg/ml)^a^	<5 pg/ml
Cre	0.24 mg/dl	Cerebral spinal fluid	
Na	122 mEq/l	IL-6 (<0.4 pg/ml)^a^	102 pg/ml
K	5.8 mEq/l	Viral culture	(–)
Cl	89 mEq/l	Protein	88 mg/dl
Glucose	55 g/dl	Cell count	2/μl
Culture		–	–
Blood	(–)	–	–
Spinal fluid	(–)	–	–
Nasal	*Haemophilus influenzae*	–	–

^a^Normal value or range. IL-6 was measured using Human IL-6 CLEIA (Fujirebio, Japan). IL-1β, IL-8, and IL-10 were measured using BIOSOURCE IL-1β, IL-8, and IL-10 ELISA kits (BioSource Europe, Nivelles, Belgium). MCP-1 and TNF-α were measured using Human MCP-1 and TNF-α Immunoassay kits (R&D Systems, Minneapolis, MN, USA)

**Fig. 1:**
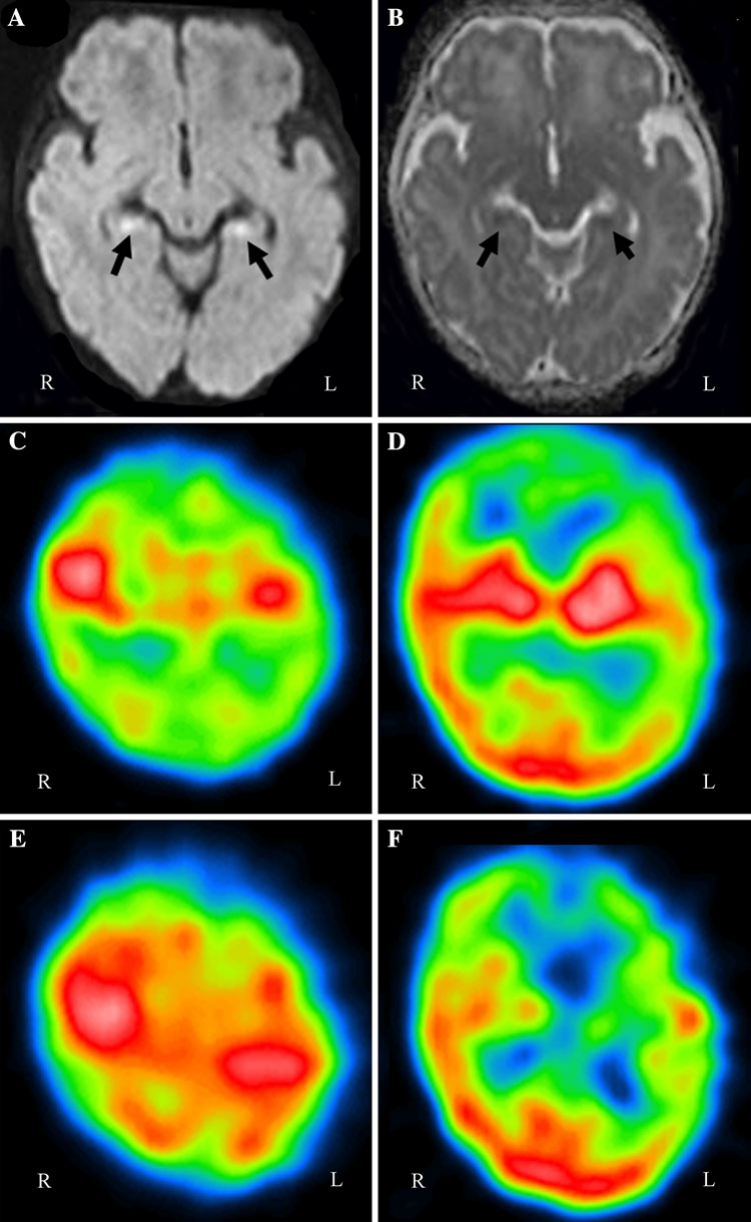
Magnetic resonance imaging (MRI) on day 6. Diffusion-weighted imaging (DWI) (**a**) revealed bilateral high-intensity regions in the hippocampus, and the apparent diffusion coefficient (ADC) (**b**) was low in the same regions. **c**–**f** ^99m^Tc- ethylcysteinate dimer (ECD) single-photon emission computed tomography (SPECT). Axial views through the parietal lobes (**c**, **e**) and through the cerebral cortex, white matter, and basal ganglia (**d**, **f**). *Upper row* (**c**, **d**): At 18 days after onset (hospital day 18), SPECT showed hypoperfusion of the bilateral frontal and parietal lobes and the unilateral temporal lobe with some predominance on the left side. *Lower row* (**e**, **f**): At 3 months after onset, hypoperfusion of the bilateral parietal lobes on SPECT had improved, but hypoperfusion of the unilateral temporal lobe with some predominance on the left side was exacerbated

The patient was intubated and ventilated immediately. Profound sedation was used because high-pressure mechanical ventilation was required. Immunomodulatory therapy with high-dose steroids (methylprednisolone 30 mg/kg/day for 3 days) was also initiated because the respiratory therapies were ineffective. On day 5, there were no significant abnormalities on T_1_-weighted, T_2_-weighted, and fluid-attenuated inversion recovery magnetic resonance imaging (MRI), but the bilateral hippocampus showed a high signal intensity in diffusion-weighted imaging (DWI) MRI with reduction of the apparent diffusion coefficient (ADC) (Fig. 1 a, b). An electroencephalogram (EEG) was normal on day 5. The patient was extubated on day 7.

On day 14, the hippocampal abnormality and ADC were improved on DWI MRI. However, on the same day, the patient presented with trembling of the upper limbs from side to side, and an EEG showed occasional unilateral spikes in the parietal area. Therefore, anticonvulsant therapy with phenobarbital was started. Serological tests for rubella, measles, cytomegalovirus, Epstein–Barr virus, herpes simplex virus, and autoantibodies associated with limbic encephalitis (antibodies against the glutamate receptor Gluε2, voltage-gated potassium channel, and NR1/NR2 subunits of the *N*-methyl-d-aspartate receptor) were negative during the acute phase. RSV antibody in serum was positive (NT ×16) on day 3. His mother had no ovarian teratoma during pregnancy.

At admission, interleukin (IL)-6 was increased markedly in the spinal fluid, and IL-6 serum levels were even more elevated compared with the spinal fluid [2, 3]. In the acute phase, serum IL-8 was significantly and markedly increased, but IL-1β, IL-10, monocyte chemoattractant protein (MCP)-1, and tumor necrosis factor (TNF)-α levels were only slightly increased or not significantly altered (Table 1). Therefore, the patient was diagnosed with encephalopathy caused by RSV infection, as suggested by a prior study showing stupor and hypotonia lasting for at least 24 h and seizure [6]. ^99m^Tc-labeled ethylcysteinate dimer (ECD) SPECT on hospital day 18 showed hypoperfusion of the bilateral frontal and parietal regions and the unilateral temporal region with some predominance on the left side (Fig. 1c, d).

Follow-up EEG, MRI, and SPECT were performed 3 months after onset. EEG was normal and phenobarbital treatment was ended. MRI did not show any signs of atrophy. ^99m^Tc-labeled ECD SPECT at 3 months still showed hypoperfusion of the bilateral frontal region and unilateral temporal region with some predominance on the left side. Hypoperfusion on SPECT had improved in the bilateral parietal lobes but persisted in the frontal and temporal regions (Fig. 1e, f). The patient has no neurological deficit at 6 months of age.

## Discussion

The present case was diagnosed as RSV encephalopathy accompanied by markedly increased levels of serum IL-6. These findings are consistent with alteration of cytokine levels in influenza virus-associated encephalopathy, because previous studies have shown marked increases of IL-6 in serum and spinal fluid in this disease [7, 8]. In particular, the serum IL-6 level has been suggested to be a useful indicator for the diagnosis and clinical severity of viral encephalopathy [7]. In our case, spinal fluid IL-6 was increased significantly and serum IL-6 showed an even greater increase. However, we also note that some studies have not found increased serum IL-6 in RSV encephalopathy [2–4].

Our observations in this case indicate that RSV encephalopathy can occur during SIRS. The cause of SIRS is essentially overproduction of inflammatory cytokines such as TNF-α, IL-1β, and IL-6 [9]. Thus, SIRS associated with RSV infection may be characterized by progression of severe pneumonia caused by hematogenous actions of elevated IL-6 on the lung and encephalopathy of the cytokine storm type resulting from similar actions of IL-6 on the brain. This suggestion is supported by the marked increase in serum IL-8, which is similar to the cytokine storm that can occur in influenza virus-associated encephalopathy [10]. The elevated IL-6 levels in the spinal fluid and serum and elevated IL-8 levels in the serum suggest that cytokine storm may be involved in the pathogenesis of RSV encephalopathy. However, the etiology of RSV encephalopathy is unclear. Specifically, it is uncertain whether direct impairment of the central nervous system is primarily responsible for the cerebral involvement in RSV disease, or if this is caused by increased production of one or more cytokines in cerebrospinal fluid during the acute phase [11, 12].

The characteristics of brain imaging findings in RSV encephalopathy are also unclear. A recent report showed that these findings often include unilateral brain hemisphere abnormalities on CT, MRI, and SPECT [3, 11, 13]. Our case exhibited hypoperfusion of the bilateral hippocampal regions and the unilateral temporal region on SPECT. In general, limbic encephalitis associated with infection is most commonly caused by herpes simplex virus, but a test for herpes simplex virus IgM antibody was negative on day 12. Most cases of autoimmune limbic encephalitis are indicated by autoantibodies, but in this case these autoantibodies were negative or normal in the acute phase [14]. Förster et al. showed that hippocampal DWI hyperintensities and a reduction of ADC on MRI were caused by limbic encephalitis with viral infection. These infections included human herpesvirus 6 and herpes simplex virus, but not RSV [15]. Therefore, this patient was diagnosed with RSV encephalopathy without limbic encephalitis.

After 3 months, hypoperfusion on SPECT had improved in the bilateral parietal lobes, but persisted in the frontal and temporal regions without neurological sequelae. Similarly, Hirayama et al. [16] found that residual cerebellar abnormalities on SPECT persisted 1 year after onset in RSV encephalitis with mild ataxia. Thus, RSV encephalopathy may be associated with prolonged cerebral hypoperfusion. Our case suggests that detection of severe RSV infection should lead to consideration of an early diagnosis of encephalopathy associated with SIRS.
